# A real-world experience of transition to robotic-assisted thoracic surgery (RATS) for lung resections

**DOI:** 10.3389/fsurg.2023.1127627

**Published:** 2023-03-15

**Authors:** Alessandro Palleschi, Giovanni Mattioni, Paolo Mendogni, Davide Tosi

**Affiliations:** ^1^ Thoracic Surgery and Lung Transplantation Unit, IRCCS Foundation Ca’ Granda Ospedale Maggiore Policlinico, Milan, Italy; ^2^ School of Thoracic Surgery, University of Milan, Milan, Italy

**Keywords:** robotic-assisted thoracic surgery, RATS, biportal, uniportal, lung cancer, brief report

## Abstract

**Objective:**

We report our experience of transition to robotic-assisted thoracic surgery (RATS) for lung resections with the da Vinci Xi surgical system, exposing short-term results.

**Materials and methods:**

This is a single-center, retrospective analysis of RATS lung resections performed between April 2021 and September 2022 during our new robotic program. The surgical approach evolved over time, starting from a four-arm approach with four incisions. Alternative RATS approaches were subsequently evaluated, such as uniportal and biportal.

**Results:**

During a 17-month period, 29 lung resections were performed. Of them, 16 were lobectomies, 7 were segmentectomies, and 6 were wedge resections. The most common indication for anatomical lung resection was non-small cell lung cancer. A uniportal approach was used for two simple segmentectomies and a biportal RATS was performed in five lobectomies and two segmentectomies. A mean number of 8.1 lymph nodes and a mean of 2.6 N2 and 1.9 N1 stations were resected during surgery, and no nodal upstaging was observed. Negative resection margins were 100%. There were two (7%) conversions, one to open surgery and one to video-assisted thoracic surgery (VATS). Eight (28%) patients experienced complications with no 30-day mortality.

**Discussion:**

High-ergonomic and high-quality views were immediately observed. After some procedures, we abandoned uniportal RATS because of the possibility of arm collisions and the necessity of a VATS-skilled surgeon at the operating table.

**Conclusion:**

RATS for lung resections was safe and effective, and from the surgeon's standpoint, several practical advantages over VATS were observed. Further analysis on outcomes will help better understand the value of this technology.

## 1. Introduction

The application of robotic surgical systems in thoracic surgery is still rapidly increasing. Robotic-assisted thoracic surgery (RATS) is believed to offer specific advantages: enhanced and 3D view, instruments articulation, higher ergonomics, and movement filtering. The transition to RATS in lung resections has been suggested to differ when starting from a precedent open surgery experience rather than starting from video-assisted thoracic surgery (VATS). The approach used (e.g., uniportal, biportal) may also have a significance. In April 2021, our thoracic surgery department started a RATS program, using the da Vinci Xi surgical system (Intuitive Surgical, California, United States). Both pulmonary and mediastinal procedures were performed. Previously, lung resections were routinely performed with a uniportal VATS approach. In this brief report, we expose our real-world experience of transition to RATS for lung resections, along with obstacles and challenges met.

## 2. Materials and methods

A single-center, retrospective analysis was conducted. Data from patients who underwent RATS lung resections with the da Vinci Xi from April 2021 to September 2022 in our institution (Fondazione IRCCS Ca’ Granda Ospedale Maggiore Policlinico of Milan, Italy) were retrieved. All patients provided informed consent prior to surgery, and the study was approved by the ethical review board of our institution (approval no. 3.11/2022-273). A dedicated weekly session was established for the RATS program. Initially, mediastinal and simple lung procedures were performed, in order to get familiar with the robotic system. The selected anatomical lung resections were lobectomies and simple segmentectomies. Pneumonectomies, bilobectomies, and sleeve lobectomies were excluded, as well as lung resections after neoadjuvant treatment. The previous diagnostic and therapeutic pathway for non-small cell lung cancer (NSCLC) was not changed by the RATS program. Prior to surgery, all patients affected by a diagnosed or suspected NSCLC received a contrast-enhanced computed tomography (CT) scan of the thorax and a total body fluorodeoxyglucose positron emission tomography (FDG-PET) scan. If a pathological diagnosis was not available, a frozen section analysis on a wedge resection was performed prior to an eventual anatomical resection. During segmentectomies, an N1 lymph node was resected for intraoperative frozen section analysis, and if positive, a lobectomy would have been performed. The intersegmental plane was identified using indocyanine green venous injection after arterial stapling. Preoperative functional tests (mainly respiratory) were performed in accordance with international guidelines (1, 2).

### 2.1. RATS approaches

**Figure 1:**
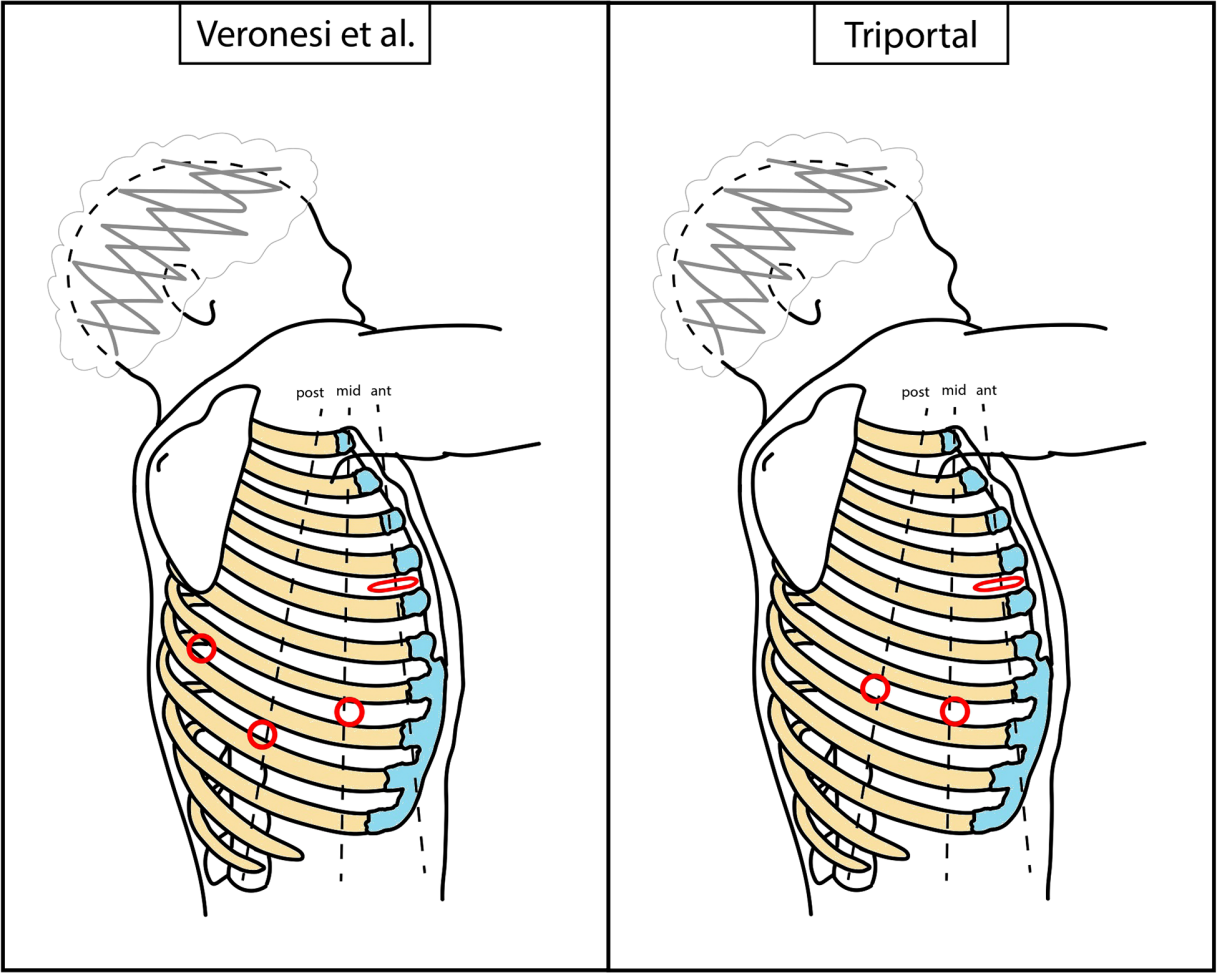
Schematic representation of RATS accesses positioning. Dimensions and distances are not to scale, but only indicative. Red circle, robotic port; red flattened circle, mini-thoracotomy (valid as assistant access too). RATS, robotic-assisted thoracic surgery**.**

**Figure 2:**
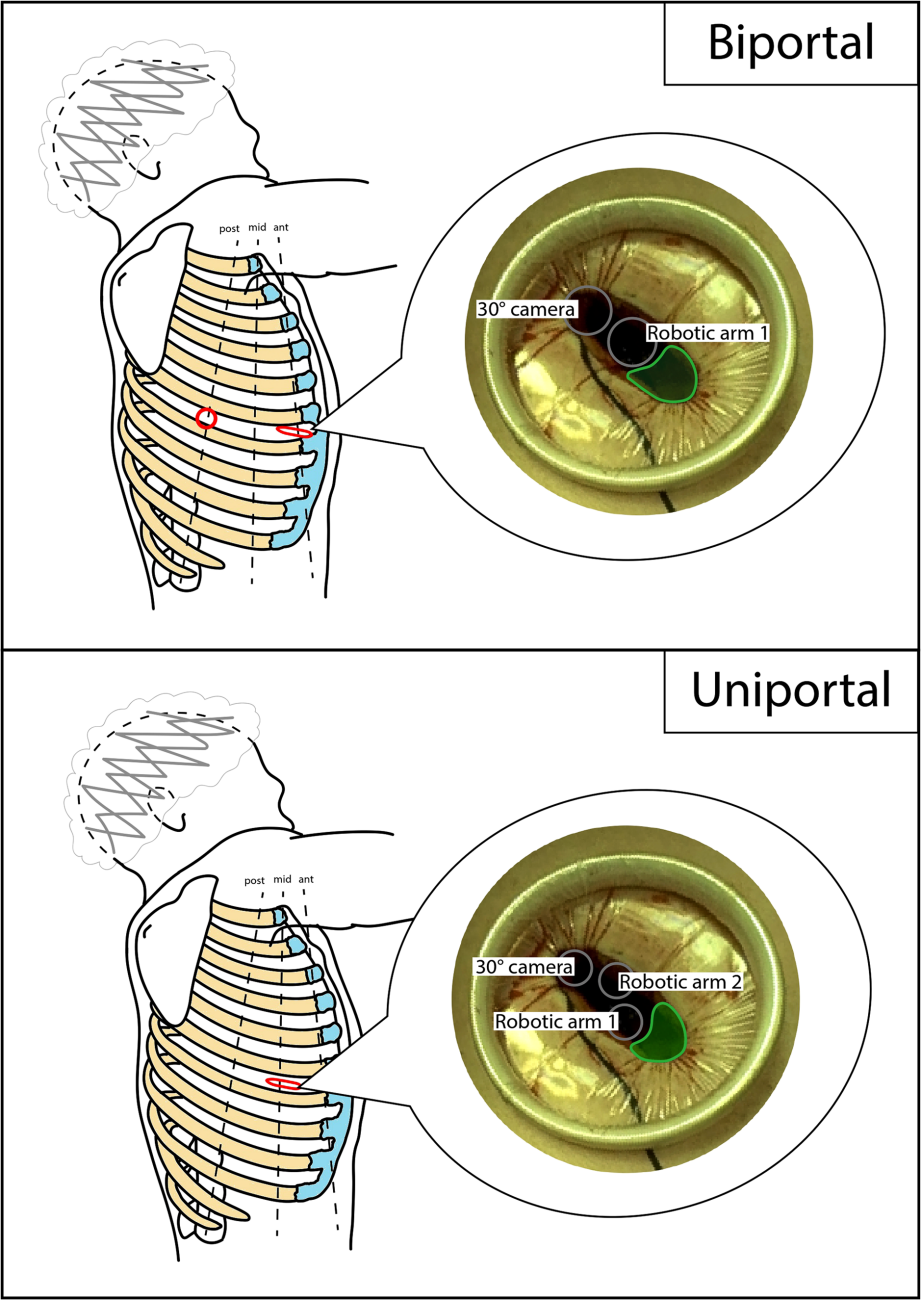
Schematic representation of biportal and uniportal RATS accesses positioning. Dimensions and distances are not to scale, but only indicative. Red circle, robotic port; red flattened circle, mini-thoracotomy (valid as assistant access too); gray circle, robotic trocar; green area, assistant area/access. RATS, robotic-assisted thoracic surgery**.**

In the early phase of the program, the robotic-assisted (RA) approach with four arms described by Veronesi et al. was employed. The anterior mini-thoracotomy, typically in the fourth intercostal space, was used for both a robotic arm and the space for the assistant activity. A soft tissue retractor (Alexis®) was positioned here. Additionally, three robotic ports were positioned along the seventh and eighth intercostal space, with the camera located in the midaxillary line port (3, 4). Subsequently, alternative RATS approaches were applied. The three-arm biportal approach consisted in a mini-thoracotomy, usually performed in the sixth to seventh intercostal space, on the anterior axillary line, and an additional robotic port positioned in the sixth to seventh intercostal space, on the posterior axillary line. The camera port, an arm port, and the space for the assistant activity were located in the mini-thoracotomy, and the other arm port was positioned in the second access. The three-arm uniportal approach was based on a 5-cm mini-thoracotomy in the sixth intercostal space, on the midaxillary line, from which both the robotic arms and the assistant could work. Finally, after this experience, a triportal approach was attempted, with a mini-thoracotomy in the fourth-fifth intercostal space, on the anterior axillary line, to accommodate both the arm port and the assistant. The camera port was positioned in the seventh to eighth intercostal space, midaxillary line, and the arm port in the seventh to eighth intercostal space. The patient position was always the same, in the lateral decubitus. A schematic representation of approaches can be found in Figures 1, 2. Manual staplers were used by the assistant. No CO_2_ was insufflated and a 30° camera was used.

## 3. Results

During a 17-month period of RATS program, 29 lung resections were performed with the da Vinci Xi robotic surgical system. Of them, 16 were lobectomies, 7 were segmentectomies, and 6 were wedge resections. All procedures were performed by two surgeons (DT and AP). The most common indication for anatomical lung resection was NSCLC. Only one hamartoma was treated with segmentectomy due to its position, impeding a wedge resection. Mean age of patients was 64 (±12) years, and 20 (69%) were female. Twelve (41%) were never smokers, whereas 11 (38%) were former smokers and 6 (21%) were active smokers. Twenty-three (79%) had at least one polymorbidity (e.g., systemic arterial hypertension, diabetes), and 10 (34%) at least two. Concerning preoperative respiratory function, mean % predicted (%p) forced expiratory volume 1 s (FEV1) was 103% (±0.21), mean %p forced vital capacity (FVC) was 109% (±0.18), and mean %p diffusing capacity of the lungs for carbon monoxide (DLCO) was 77% (±0.16). Details concerning disease and procedure characteristics and perioperative outcomes are reported in Table 1. Mean operative time was 238 min (median 232) for lobectomy, 230 min (median 212) for segmentectomy, and 98 min (median 99) for wedge. Even if a formal statistical analysis was not conducted (given the small number of procedures), a difference in operative times between the different approaches of lobectomy was noted. In particular, the biportal lobectomies carried an additional mean of 101 min than multiport lobectomies. There were two conversions (7%). One was a planned RATS segmentectomy for a cT1cN0 stage NSCLC that was converted to VATS lobectomy for technical reasons. The other one was a RATS lobectomy in a cT3N1 stage NSCLC that was converted to open surgery due to bleeding from a pulmonary artery branch. Regarding lymphadenectomy, we found that a mean number of 8.1 lymph nodes were retrieved during surgery. A mean number of 2.6 N2 and 1.9 N1 stations were resected. No nodal upstaging was observed. Negative resection margins were obtained in all cases (100%). The mean postoperative length of stay was 6.8 days for lobectomy, 7.2 for segmentectomy, and 3 for wedge resection. Mean chest tube duration was 5.3 days for lobectomy, 5.7 for segmentectomy, and 1.8 for wedge resection. Globally, eight (28%) patients experienced complications. Of these, six were grade I and three were grade II [Clavien–Dindo classification (5)]. Further details are available in Table 2. No 30-day readmission in hospital nor 30-day death were recorded.

**Table 1:** Surgical details of patients that underwent RATS lung resections.

Variable	Value
Histotype^a^	
Adenocarcinoma	20 (87%)
Squamocellular carcinoma	1 (4%)
Large cell anaplastic carcinoma	1 (4%)
Hamartoma	1 (4%)
pTNM stage 8th ed. (*n* = 22)^a^	
0 (is)	1
IA1	3
IA2	6
IA3	2
IB	4
IIA	1
IIB	3
IIIA	2
Tumor location^a^	
Right upper lobe	10
Right inferior lobe	4
Left upper lobe	2
Left S6 segment	3
Left S1–S3 segments	2
Right S1–S2 segments	1
Right S3 segment	1
Conversions^a^	
RATS to open surgery	1
RATS to VATS	1
Lymphadenectomy^a^	
Mean no. of resected lymph nodes	8.1
Mean no. of resected lymph node stations	
N1	1.9
N2	2.6
Nodal upstaging	0%
Negative resection margins	100%
Mean operative time (min)^b^	
Lobectomy	238
Segmentectomy	230
Postoperative complications	
Grade I	6
Grade II	3
Mortality	0%
Mean hospital length of stay (days)	
Lobectomy	6.8
Segmentectomy	7.2
Wedge resection	3
Mean chest tube duration (days)	
Lobectomy	5.3
Segmentectomy	5.7
Wedge resection	1.8

RATS, robotic-assisted thoracic surgery**.**

^a^ Concerns only RATS anatomical lung resections.

^b^ Concerns only RATS anatomical lung resections without conversion.

**Table 2:** Early postoperative complications details.

Cases (*n* = 8 pts)	Grade I	Grade II
2° lobectomy	Dyspnea	
4° segmentectomy	PAL, subcutaneous emphysema	PAL (blood patch)
5° segmentectomy		Pneumonia (antibiotics)
8° lobectomy		
10° lobectomy	PAL	
11° lobectomy	TIA	
7° segmentectomy	PAL, subcutaneous emphysema	
15° lobectomy	PAL, subcutaneous emphysema	Anemia (transfusion)

pts, patients; PAL, prolonged air leak; TIA, transient ischemic attack.

The first lobectomy was performed after 8 procedures with a standard four-arm approach, whereas the first segmentectomy after 21 cases and with a uniportal approach. Overall, four uniportal (two segmentectomies and two uniportal pleurodesis with wedge resection), and eight biportal (five lobectomies, two segmentectomies, and one pleurodesis with wedge resection) procedures were performed. The first uniportal operation was a pleurodesis and wedge resection, whereas the first biportal procedure was a simple segmentectomy.

## 4. Discussion

Our study represents a real-world report of a thoracic surgery unit transitioning to RATS for lung resections. During a transition to a new technique or approach, it is legit to question if it will achieve better results. Four major objectives should be pursued: higher, or at least equal, safety, reduced time, lower costs, and increased results. In this case, increased results concerns both surgical and oncological outcomes. However, all these four may not be always obtained simultaneously. In the case of lung resections, the transition to RATS can happen either from open surgery or VATS. Differences between these two transitions are thought to exist. It has been suggested that transition from open surgery to RATS is easier than from open surgery to VATS (4). The hypothesized reason is the similarity of surgical steps in lung resections between open and RATS. However, it is also common to believe that an experienced VATS surgeon has less difficulties in approaching RATS than an open surgeon. This idea could be supported by the similarity of RATS to VATS because of the reduced to absent tactile feedback and the visualization of the thoracic cavity through the screen (6). Results from single-surgeon experiences of transition to RATS suggest similar performances between RATS and VATS. Initially, RATS operative times are longer probably due to docking time and familiarization with instruments (7, 8).

At the beginning of our RATS experience, we had some concerns on performing multiple accesses for a robotic lung resection, rather than a single one as in uniportal VATS. However, after some operations, we acquired confidence with the multiport approach. Some practical advantages over VATS were immediately observed after the first procedures. First of all, the high ergonomics resulted in a less tiring and more comfortable surgery for the console operator. In addition, the quality of the view was significantly higher, thanks to the enhanced quality of the video, the 3D vision, and the stability of the camera. This somehow helped compensating the absence of haptic feedback, especially during dissection of hilar elements. As a consequence, we expected that RATS would result in a higher number of resected lymph nodes than VATS. Nevertheless, even if a formal analysis and comparison were not made, the results did not favor this hypothesis. We are still in an early phase and more cases are needed to make our results more robust. In our experience, staplers were used by the assistant, thus reducing the independence of the console operator. However, we believe autonomy was higher compared to VATS, given that all the instruments, camera included, were easily controlled by the operating surgeon.

Our previous uniportal VATS experience eventually led our team to experiment alternative RATS approaches, with the objective of reducing the incisions. Therefore, both biportal and uniportal RATS approaches were performed. The time required for setting the robotic arms, and for adjusting them during the operation to avoid collision, inevitably determined longer operative times. Collisions were significantly higher with the uniportal approach, and as reported in recent papers, it required the presence of a uniportal VATS-skilled surgeon at the operating table (9). Collisions between instruments are potentially harmful for the patient, and the assistant's help revealed to be important during several steps. Given these issues, we decided to abandon the uniportal approach for major lung resections. In addition, it should be kept in mind that to date this type of technique is not approved by the manufacturer, so medical–legal issues could also arise in case of major complications. We believe that in the future, once the technology for the uniportal approach is developed, this may be a viable option under conditions of greater patient safety.

At present, our preferred approach for RATS lung resections is the triportal one. In general, a certain degree of freedom of choice on the number and location of the incisions is accepted, based on surgeon's preference and case characteristics. No superior study between one approach and another has yet been published. Still, one interesting issue is multiple nerve damage as a possible cause of more pain. Some authors believe this may cause more pain compared to approaches that are performed accessing only one or two intercostal spaces (10–12). In our experience, multiport VATS was thought to be more painful than uniportal VATS (13). During our RATS program we did not systematically collect quality data concerning postoperative pain; thus, we were not able to make any comparison or analysis. From a theoretical standpoint, damaging only one intercostal nerve rather than more than one, when positioning multiple ports, would logically result in less pain. However, to date, a reliable systematic analysis and comparison is still not available and is likely to be particularly complex given the number of factors involved in postoperative pain. On the other hand, our experience taught us that practical advantages of accessing the thorax through different intercostal spaces are ensuring more possible directions for instruments and a wider triangulation.

In our experience, we preferred not to use CO_2_ insufflation. We considered that the benefit of CO_2_ in lung resections was not worth the need for dedicated devices (e.g., Alnote-Lapsingle©), given the presence of the mini-thoracotomy. We believed it would be probably simpler to use CO_2_ with a robotic portal (RP) approach (3). Of course, we are aware that CO_2_ pressure would result in a better exposure of structures, mainly by compression of lung and diaphragm. In fact, during our thymic RATS procedures, CO_2_ insufflation was routinely used, thanks to the application of an RP approach.

We believe that RATS is an interesting technology that may be beneficial in lung resections. However, given its cost, it is expected to bring benefits not only to surgeons but also to patients in order to be justified. Thus, we will monitor outcomes of RATS procedures in our center. It may reveal to perform better in determined surgical gestures, as suturing. In fact, it resembles the open surgery experience, and this may facilitate procedures as sleeve resections, as reported by several authors (14–16). Thus, the positive impact of RATS may appear more significant in this kind of procedures, rather than in routine ones.

Some limits of this study can be identified. First, the cohort of patients is small and from a single center, limiting the power of our results. In addition, we are still in the learning curve phase, thus requiring more time to produce definitive data from both involved surgeons.

## 5. Conclusion

We found that RATS lung resections were safe and effective, and from the surgeon's standpoint, several practical advantages over VATS were observed. Results are probably premature to be correctly interpreted and, of course, we are still in the learning curve phase. At present time, we believe that the uniportal approach is not advisable because of possible conflicts between the robotic arms and the resulting risks to the patient. Further analysis of outcomes will help better understand the value of this technology.

## Data Availability

The raw data supporting the conclusions of this article will be made available by the authors, without undue reservation.
